# Lead Removal from Contaminated Shooting Range Soil using Acetic Acid Potassium Chloride Washing Solutions and Electrochemical Reduction

**DOI:** 10.5696/2156-9614-7-13.22

**Published:** 2017-03-29

**Authors:** Effiong Ukorebi Etim

**Affiliations:** Department of Chemistry, University of Ibadan, Ibadan, Nigeria

**Keywords:** soil, lead, electrochemical reduction, soil washing, ex-situ batch testing

## Abstract

**Background.:**

Cleanup of soils contaminated with toxic metals is a difficult task due to the method inefficiency and the destructive nature of clean-up techniques on soil ecosystems.

**Objectives.:**

This study was performed to improve the removal efficiency of an acetic acid washing solution for the removal of lead (Pb) from soil. Acetic acid was used in combination with different concentrations of potassium chloride. In order to maximize the removal of Pb from the leachate, different electrode combinations were applied to the washing solutions.

**Methods.:**

Acetic acid/potassium chloride washing solutions and electrochemical reduction were applied to lead-contaminated soil obtained from an impact berm of a major military shooting range in Ibadan, southwestern Nigeria. The soil was subjected to 5% acetic acid/5% potassium chloride (KCL) and 5% acetic acid/10% KCL solutions in an ex-situ batch experiment. The leachate was electrochemically reduced using 12 volt direct current with a current of 7 amps and 2.5 amps, with aluminum (Al)-Al, iron (Fe)-Fe, Al-Fe and Fe-Al electrodes.

**Results.:**

The 5% acetic acid/5% KCL proved more efficient for Pb removal in soil with values ranging from 74.9% to 86.9% for 3% soil pulp densities with one washing time of 6 hours. Removal efficiency of Pb from the contaminated soil significantly decreased as the soil pulp density increased. The Al-Al and Al-Fe bipolar electrode combinations showed better removal efficiency of Pb from the leachates with values of 93.7% and 95.6% for 7 amps and 94.5% and 97.3% for 2.5 amps, respectively.

**Conclusions.:**

The combined 5% acetic acid and 5% potassium chloride washing solution enhances the removal efficiency of Pb in soil and poses less risk to the soil ecosystem and the environment in general.

## Introduction

Soil contaminated with lead is a major concern all over the world. This is due to its persistence and toxicity. Lead is known to cause health problems such as cognitive impairments, behavioral disorders, stroke and even death.^1^ Different soil decontamination methods have been tried such as physical, chemical/soil washing, electrochemical, biological and integrated processes that combine different methods.^2^ Among these methods, soil washing (*ex situ or in situ*) has been one of the most useful treatments because of its rapid efficient remediation process and its low cost in comparison to other remediation techniques.^3–6^ Several washing solutions for extracting metal ions have been widely used and generally include acids, bases and chelating agents.^7^ Strong acids and chelating agents such as hydrochloric acid, nitric acid, sulfuric acid and ethylenediaminetetraacetic acid (EDTA) have been proven effective for metal extraction. However, strong acids at high concentration are lethal to soil micro-flora and destructive to the physical and chemical properties of soil due to mineral dissolution.^8^ In addition, EDTA is expensive and often results in generation of metal ions in the solution phase which require further treatment to remove.^9^,^10^ A combination of acids at low concentrations with different salts like sodium chloride and calcium chloride have shown promising results in lead removal efficiency. In one instance, lead removal was reported to be 75% from shooting range soil by leaching with 1 M sulfuric acid and 4 M sodium hydroxide, along with removal of copper, antimony and zinc.^11^ In another experiment, 0.05 M sodium-ethylenediaminetetraacetic acid was used to extract lead from soil at a 1:10 soil to liquid ratio, and this resulted in a removal efficiency of 50–70%.^12^ Another investigation utilized a 0.01 M EDTA leaching solution to remove lead from soil and achieved a 46–55.7% removal efficiency, whereas a different investigation used a 5.5 mol sodium chloride (NaCl) solution at pH 3.0 to leach 65% of the lead from soil.^9^,^13^ Acetic acid can form relatively strong complexes with metal ions and is easily biodegradable and environmentally friendly, but it presents a lower effectiveness in the removal of metal ions with values sometimes below 45%.^14^,^15^ The objective of this investigation was to improve the extraction efficiency when using acetic acid solutions to leach lead from contaminated soil.

Effective soil washing produces a complex leachate solution that contains metal ions, and in order to remove the metal ions from the leachate, the leachate requires treatment. Secondary treatment like nanofiltration and electrochemical treatment to remove leached metal ions from leachates have been used.^16^ Other methods for water and waste water treatment include electrocoagulation/floatation.^17–20^ Electrochemical treatment can be efficient and cost effective for removing metal ions from supernatant solutions and can also be used for metal recovery from contaminated soils through electrolytic recovery and electro-deposition.^13^,^16^,^21^

This study was performed to improve the removal efficiency of an acetic acid washing solution for the removal of lead from soil. Acetic acid was used in combination with different concentrations of potassium chloride. In order to maximize the removal of lead from the leachate, different electrode combinations were applied on the washing solutions.

**Abbreviations**
*AAS*: Atomic absorption spectrophotometer
*Al*: Aluminum
*Cu*: Copper
*EDTA*: Ethylenediaminetetraacetic acid
*Fe*: Iron
*KCL*: Potassium chloride
*NaCl*: Sodium chloride
*Pb*: Lead
*Zn*: Zinc

## Methods

### Soil Sampling and Analysis

**Figure 1:**
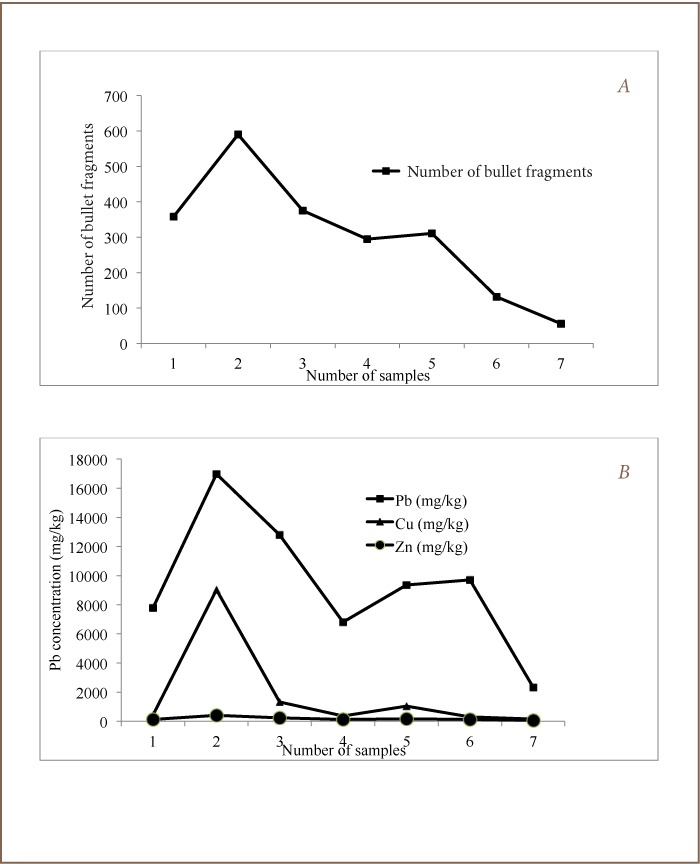
Relationship between soil metal levels and corresponding number of bullet pellets in soil (A) Number of bullet fragments (B) Metal concentration.

Lead contaminated soil was obtained from an impact berm of a major military shooting range in Ibadan, southwestern Nigeria. A systematic sampling procedure was adopted for the impact berm. The impact berm measured about 100 m in length and 18 m in width, with an estimated area of 1800 m^2^. It consisted of a front slope that was directly impacted by bullet pellets from shooting activities and back slope. The front slope of the berm was segmented into seven sampling areas with a length of about 14 meters each. At each sampling area, three contaminated top soil samples (0–15 cm in depth) were randomly (about 10 meters apart on a triangular pattern) collected and mixed together to form a single bulk sample. A total of seven contaminated top soil samples (25 kg each) were collected in plastic bags and transported to the laboratory where they were air-dried and crushed and sieved through a 4 mm mesh sieve. A 20 kg portion each of these seven sieved contaminated top soil samples were thoroughly mixed together to obtained a composite sample. Furthermore, a portion (20 kg) of this composited soil was set aside while the remainder was fractionated to 2.00, 0.85, 0.425, 0.250, 0.180 and 0.150 mm particle sizes aggregate using corresponding mesh size sieves. In addition, the total number of bullet pellets found within each of the seven top soil samples were sorted and quantified before sample pretreatment. The corresponding numbers of bullet pellets in the seven soil samples were 358, 590, 375, 295, 311, 132 and 56, respectively, and were between 1.45 to 10.7 grams (*Figure 1A*). The berm soil properties (pH-6.78 ± 0.26; % sand-63.3 ± 9.2; % clay-11.4 ± 4.9; % silt-25.4 ± 5.8; % organic matter-3.03 ± 2.09) were previously reported using standardized methods.^22^ A speciation study was carried out on the seven contaminated top soil samples using sequential extraction proposed by Tessier.^23^ The exchangeable fraction was extracted with magnesium chloride solution at pH 7.0, while carbonate fraction was extracted by leaching with 1 mol L^−1^ sodium acetate which had been adjusted to pH 5.0 with acetic acid. The reducible fraction was extracted with 0.04 mol L^−1^ hydroxylamine -hydrogen chloride in 25% (v/v) acetic acid. The oxidizable fraction was determined by treatment of the sample with 30% hydrogen peroxide adjusted to pH 2.0 with nitric acid, while the residual fraction was digested with a 2:1 mixture of concentrated hydrofluoric (48% AnalaR) and perchloric acids (70% AnalaR).^24^,^25^ Total lead, copper and zinc were analyzed for the initial seven soil samples (*Figure 1*), composited soil, 2.00, 0.85, 0.425, 0.250, 0.180 and 0.150 mm fractions (*Table 1*) using an atomic absorption spectrophotometer (AAS) Buck Scientific, model 200A after digesting 2.0 g of soil with 20 mL aqua-regia for 3 hours in a hot water bath and final extract volume made to 25 mL using a standard volumetric flask.^26^ The concentration of Pb, copper (Cu) and zinc (Zn) was calculated using the formula: concentration from instrument (mg/L) x extract volume (mL)/weight of soil used for extraction (g). The composited soil, 0.850 and 0.150 mm fractions were subjected to the lead leaching experiments, and the combined leachates obtained from these lead leaching experiments were treated using the electrochemical reduction method and electrodes mentioned above.

**Table 1:** Heavy Metal Concentrations (mg/kg) in Impact Berm Soils

Sample code	Pb	Cu	Zn
n (1–7)	9397±4624 (2333–16976)	1810±3210 (157–9024)	176±117 (57.4–413)
Composite	24499	753	183
2.00 mm	23138	660	116
0.85 mm	15610	1054	150
0.425 mm	13464	529	96.0
0.250 mm	13329	668	124
0.180 mm	13679	631	132
0.150 mm	13245	594	129

n: metal concentrations in all seven soil samples

### Chemical Washing Experiment

A washing procedure was applied to the composited soil, 0.850 and 0.150 mm fractions, in order to investigate which washing solutions are most effective and to determine the effect of different concentrations of potassium chloride on the effectiveness of acetic acid for leaching Pb from the contaminated soil. In the batch washing experiment, 15, 30, 45, 60, 75 and 90 grams of the soil samples (composite, 0.850 or 0.150 mm) was weighted into six separate plastic bottles (in duplicate). Into each of these bottles was added 100 mL 5% KCL and 400 mL 5% acetic acid to give a total volume of 500 mL of washing solution. This weight/volume represents 3, 6, 9, 12, 15 and 18% soil pulp density (e.g., 90 g/500 mL × (100%) = 18%) and the batch tests were coded S3, S6, S9, S12, S15, and S18 respectively (*Table 2*). The bottles were agitated using an end-to-end (Edmund Buhler SM 25) mechanical shaker for 2 hours, after which the mixtures were filtered using Whatman (Cat No 1001, 110 mm) filter paper. The soil residue in the filter paper was then washed with deionized water sequentially twice and both supernatants from the initial leaching and washing were combined together and stored for analysis. The agitation process was repeated for 4, 6, 8 and 10 hours intervals on other sample bottles with the same weight/volume of soil and solution and with the same volume of 100 mL 5% KCL and 400 mL 5% acetic acid washing solution. The entire procedure was repeated using a different washing solution of 100 mL 10% KCl and 400 mL 5% acetic acid following the same weight/volume and interval agitating for 2 to 10 hrs. The supernatants were analyzed directly for Pb using AAS (same instrument mentioned above after calibrating using standards) and the percentage removal efficiency of Pb calculated using Equation 1 [e.g., composite soil of S3 and 2 hours duration: First leachate: (384 mg/L × 0.5 L)/(24499 mg/kg × 0.015 kg) = 52.2%. Duplicate leachate: (373 mg/L × 0.5 L)/(24499 mg/kg × 0.015 kg) = 50.8%. The average value was 51.5%. The percentage difference between the duplicated tests was 1.4%. Each batch washing experiment was performed in duplicate. Split samples were incorporated for instrument data validation. A t test showed no significant differences in actual and slit results. Analar grade reagents were used for the experiment.

**Table 2:** Average Percentage Removal Efficiency of Lead in Fractionated Berm Soils

Sample code/time	5% acetic acid and 5% KCL			5% acetic acid and 10% KCL		
**2 hours**	Composite	0.85 mm	0.150 mm	Composite	0.85 mm	0.150 mm
S3	51.5	50.5	56.7	35.5	26.4	55.1
S6	54.3	52.1	58.7	32.6	26.4	56.5
S9	43.9	49.2	42.1	26.7	20.3	37.2
S12	45.1	49.2	31.6	25.8	20.9	41.0
S15	34.5	47.8	37.3	22.9	18.2	29.2
S18	32.6	39.8	33.2	20.5	18.1	24.0
**4 hours**						
S3	61.7	63.7	54.1	45.0	36.5	59.6
S6	60.5	66.0	56.5	43.2	27.3	54.9
S9	50.4	53.2	48.0	36.8	24.6	47.6
S12	46.5	54.6	44.4	29.9	23.0	42.7
S15	48.1	54.3	39.3	26.7	23.3	41.6
S18	44.7	50.2	38.0	16.0	21.6	35.1
**6 hours**						
S3	74.9	86.9	77.8	46.0	43.4	54.3
S6	74.1	70.5	71.2	35.4	31.3	55.2
S9	62.3	69.0	69.5	36.1	30.0	54.0
S12	56.4	65.1	47.9	35.0	29.7	47.6
S15	56.4	66.0	42.1	27.1	29.8	43.7
S18	51.2	52.0	42.5	28.2	27.7	50.3
**8 hours**						
S3	73.9	70.8	64.4	53.4	58.3	57.9
S6	65.9	74.1	61.9	47.7	59.1	57.1
S9	65.9	74.1	60.3	46.3	46.5	49.2
S12	61.5	68.7	46.6	40.1	48.1	48.8
S15	33.6	37.5	35.9	32.5	28.6	40.9
S18	40.0	26.3	44.4	31.0	27.4	42.5
**10 hours**						
S3	64.8	71.3	66.5	64.1	69.3	68.9
S6	70.4	71.4	66.6	64.2	61.9	81.3
S9	60.8	66.3	61.3	57.7	59.1	55.2
S12	54.6	62.3	51.5	50.3	52.1	50.1
S15	48.8	62.0	46.1	43.5	49.9	35.3
S18	46.5	59.0	45.1	41.0	42.5	31.0

The percentage removal efficiency of Pb from soil samples (either composite, 0.85 or 0.150 mm) was calculated using the equation proposed by Wuana et al.^24^

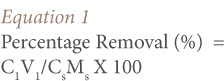



Where, C_1_ and C_S_ are the concentrations of Pb in the supernatant (mg/L) and soil sample (mg/kg) determined using AAS, respectively. In addition, V_1_ is the volume of supernatant (L) and M_S_ is the weight of the soil (kg) used for the washing experiment. C_S_: Composite-24499 mg/kg, 0.85 mm-15610 mg/kg and 0.150 mm-13245 mg/kg. V1: 0.5L. M_S_: 0.015, 0.030, 0.045, 0.060, 0.075 and 0.090 kg.

### Electrochemical Reduction Experiment

To evaluate the efficiency of the electrochemical reduction of Pb ions in the washing solutions, the reduction was carried out in the combined washing solutions using aluminum (Al)-Al, iron (Fe)-Fe, Al-Fe and Fe-Al bipolar electrodes. During the experiment, 100 ml of the combined leached solution was placed in a 250 mL glass beaker. A pair of electrodes, 14 cm long and 2 cm in diameter, were placed 5 cm apart inside the solution. The set up was subjected to electro-analytical measurement using the bipolar electrodes with a 12 volt direct current power source with current intensities of 7 amps and 2.5 amps for 5, 10, 15, 20, 25 and 30 minutes. Before and after the electrochemical reduction process, 10 ml of the electrolyte was directly analyzed for Pb by AAS.

Removal efficiency of Pb was calculated by the equation:

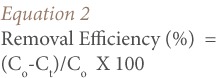



Where C_0_ is the initial Pb concentration (mg/L) before electrochemical reduction and C_t_ is the Pb concentration (mg/L) after the experiment at a given time interval.

## Results

### Lead Levels in Soil

According to previous studies, the berm soil is reducing in nature (pH 6.78±0.26) and contains high percentage sand (63.3±9.2%) and organic matter (3.03±2.09%) content.^22^ Table 1 shows concentrations of Pb, Cu and Zn obtained from the seven soil samples within the impact berm and their distribution in the various particle sizes. It was generally observed that average levels of Pb in each soil sample were much higher than Cu and Zn, a trend consistent with previously reported studies around the impact berm of shooting ranges.^22^,^25^ This trend was similarly reflected in the composited soil and its fractionated particle sizes. The concentrations of Pb, Cu and Zn in all seven soil samples varied within the range of 2333–16976, 157–9024, 54.7–413 mg/kg, respectively. The high Pb levels in the berm soil can be attributed to the presence of typical bullet fragments. Bullet fragments are alloys that contain about 93% Pb.^25^ The number of corroded bullet pellets quantified at each sample locations related positively to the concentrations of Pb, Cu and Zn in the soil (*Figure 1A and B*). In the analysis of fractionated particle sizes of the composited soil, it was observed that Pb levels varied significantly, with the composite (24499 mg/kg) and 2.00 mm particle size (23138 mg/kg) having the highest levels, while the other five particle sizes (0.85, 0.425, 0.250, 0.180 and 0.150 mm) also had relatively high, but similar levels. Levels of Cu and Zn were about the same for the various soil particle sizes with ranges of 529–1054 mg/kg for Cu and 96–183 mg/kg for Zn. From the speciation study (*Table 3*), Pb and Cu levels were more predominant in the carbonate and organic fraction, which is similar to an earlier reported study carried out within the entire range.^22^ Zinc was within the exchangeable and organic fractions.

**Table 3:** Heavy Metal Speciation (% in Geochemical Phases) of Impact Berm Soils

Fractions	Pb	Cu	Zn
Exchangeable metals	7.70	7.75	20.63
Metals bound to carbonates	18.25	25.77	3.40
Metals bound to Fe-Mn oxides	9.45	10.03	9.14
Metals bound to organic matter	31.00	24.97	13.01
Residual metals	33.60	31.47	53.81

### Lead Removal in Soil by Washing

**Figure 2:**
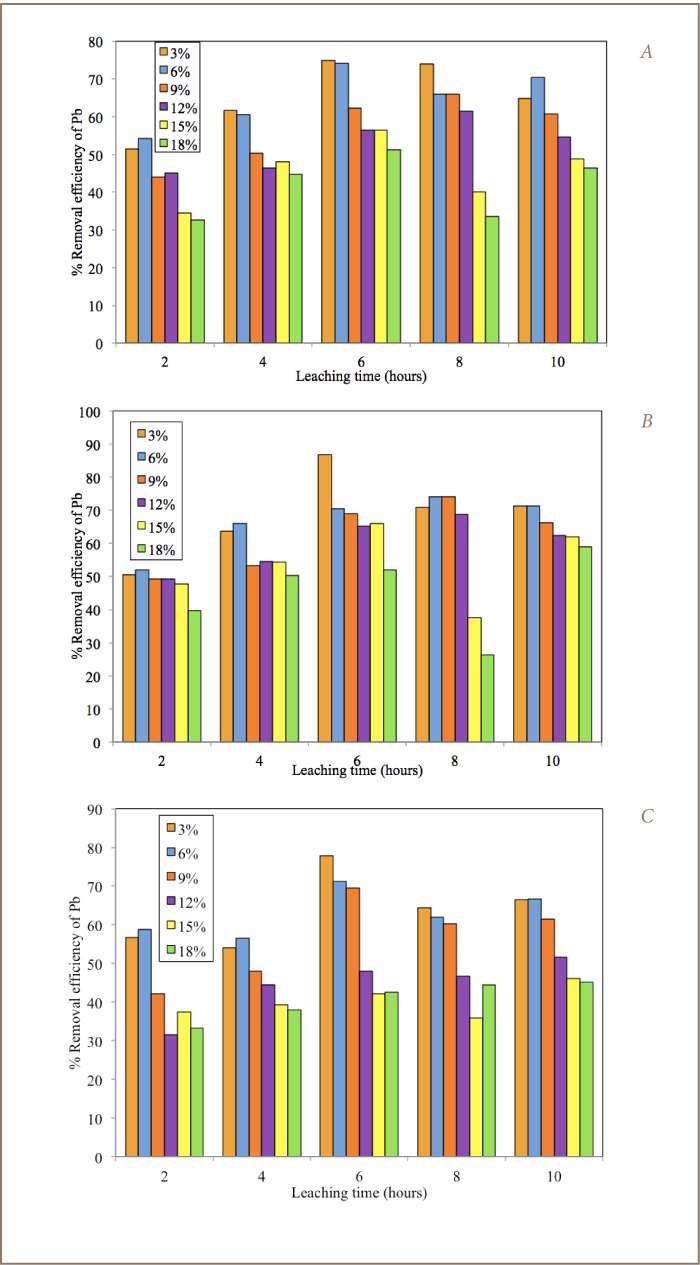
Percentage removal efficiency of Pb in fractionated berm soils using 5% acetic acid and 5% KCL washing solutions (A) composite (B) 0.850 mm (C) 0.150 mm.

**Figure 3:**
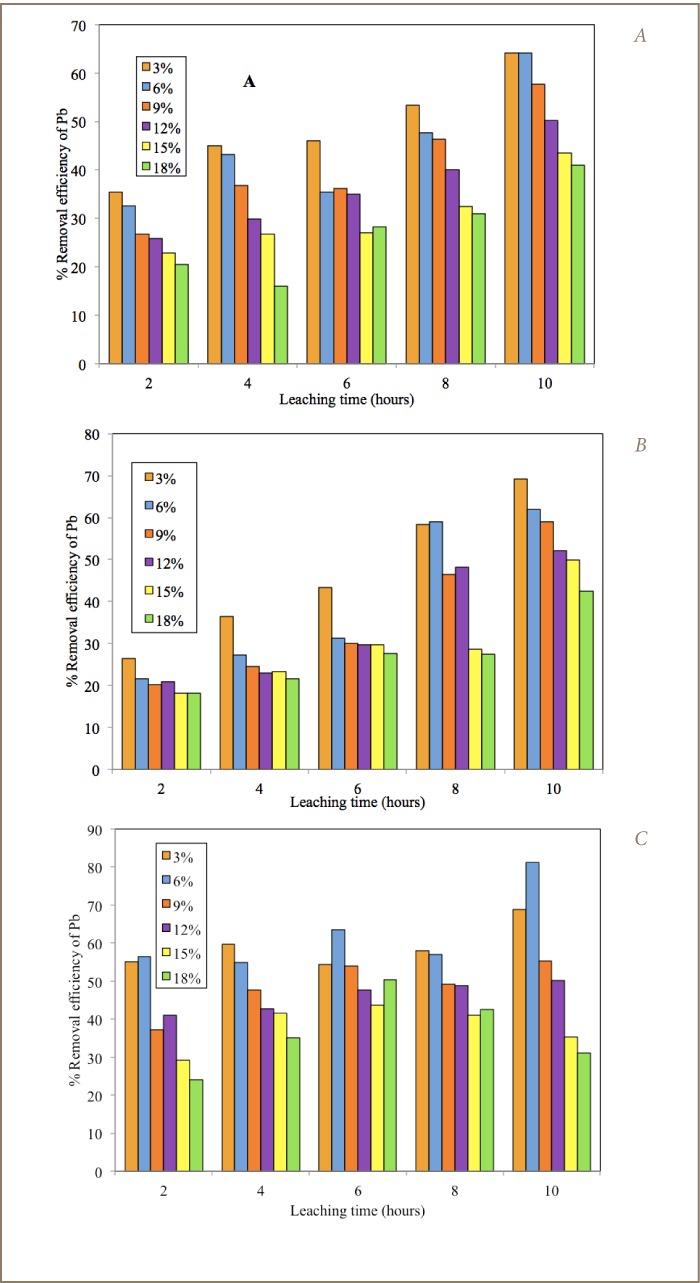
Percentage removal efficiency of Pb in fractionated berm soils using 5% acetic acid and 10% KCL washing solutions (A) composite (B) 0.850 mm (C) 0.150 mm.

Table 2 shows the average percentage removal efficiency of Pb in the composited and fractions of the lead-contaminated soils using 5% acetic acid/5% KCL and 5% acetic acid/10% KCL washing solutions as calculated using Equation 1. The calculated percentage removal efficiencies were the average values of the two batch tests. The percentage difference between the duplicate tests ranged between 1 to 3% (example of 1.4% is given above). As shown in Table 2, washing the soil using 5% acetic acid and 5% KCL solution resulted in Pb removal efficiencies between 31.6–86.9%. The best removal efficiencies for this washing solution were 74.9%, 86.9% and 77.8% for 3% pulp density at a washing time of 6 hours for the composite, 0.85 mm and 0.150 mm fractions, respectively. It was observed that pulp densities of 3, 6 and 9% were efficient for Pb removal with values sometimes greater than 60%, when washed for a duration of 4 hours and greater for the composite and 0.85 mm soil fractions. The 0.150 mm fraction only showed >60% Pb removal efficiency from 6 hours above for 3%, 6% and 9% pulp density. Longer washing time appears to improve Pb removal efficiency from contaminated soil. Generally, the removal efficiency of Pb decreased with increasing soil pulp density (*Figure 2*). However, the removal efficiency of Pb for 5% acetic acid and 10% KCL was lower than for 5% acetic acid and 5% KCL washing solution. The removal efficiencies in this washing solution ranged from 16.0–69.3%. The highest values of 64.1%, 69.3 and 68.9% were recorded for 3% soil pulp density and 10 hour duration interval for the composite, 0.85 mm and 0.150 mm fractions, respectively. The 2, 4, 6 and 8 hours washing durations for the 3–18% soil pulp densities all recorded values below 60% (*Table 2*). It was also observed that increasing soil pulp density, irrespective of washing duration, corresponded to a decrease in the percentage removal efficiency of Pb. Meanwhile, for each soil pulp density, the percentage removal of Pb increased with duration of washing time (*Figure 3*).

### Lead Removal by Electrochemical Reduction

**Figure 4:**
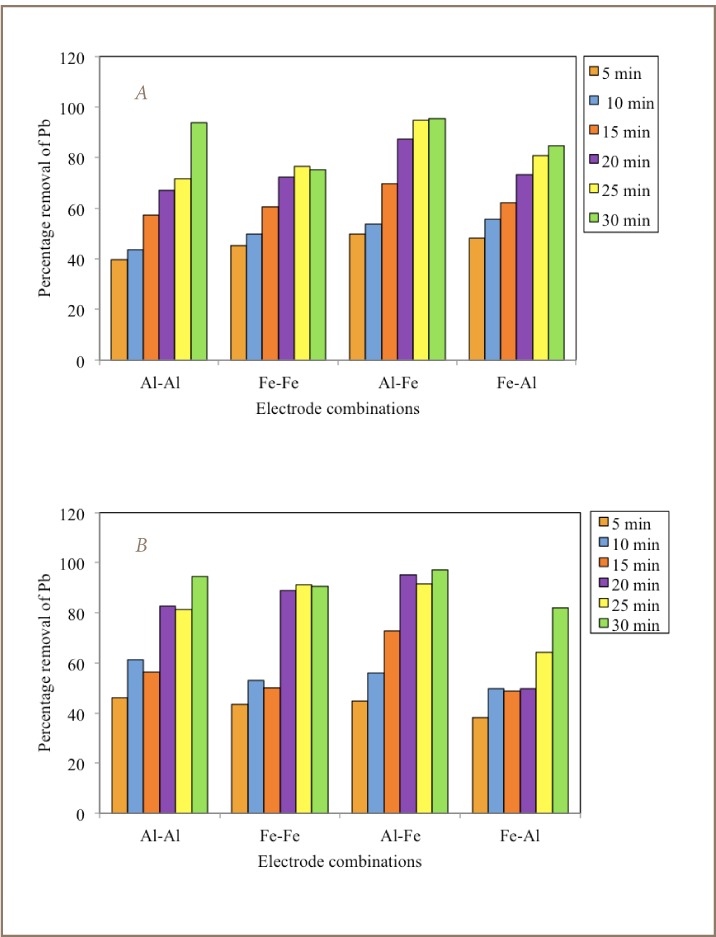
Percentage removal of Pb after electrochemical reduction using 12 volt battery with (A) 7.0 A (B) 2.5 A current.

The results of the electrochemical reduction of Pb from the combined washing solutions using different electrode combinations are illustrated in Figure 4. The removal efficiency of Pb from the combined washing solutions by electrochemical reduction was calculated using Equation 2. Compared with the initial Pb concentration, it was generally observed that the percentage removal of Pb increased with time with the majority of the highest values attained after 30 minutes. Both 7 A and 2.5 A current applications showed a similar trend in percentage removal of Pb, with values ranging between 39.7 to 95.6% and 38.1 to 97.3%, respectively, for the same electrode configuration. The Al-Al and Al-Fe bipolar electrode combinations showed better removal efficiency of Pb with highest values of 93.7% and 95.6% for 7 A and 94.5% and 97.3% for 2.5 A, respectively.

## Discussion

### Lead Removal Efficiency in Soil

The presence of corroded bullet fragments resulting from abrasion and weathering could account for the high lead concentrations in the soil fractions. Bioavailable fractions of Pb in the soils could also pose a significant environmental threat considering the high concentration of lead.^27^ Analysis of variance at an error probability of 0.05 was used to statistically evaluate the reproducibility and efficiency of each washing solution using Microsoft Excel (Redmond, WA, USA). This was done by multiple comparisons of Pb removal efficiency of the composite, 0.85 mm and 0.15 mm soil fractions for 3 to 18% soil pulp densities at each washing time of 2 to 10 hours (*Table 4*). This test showed no significant difference in percentage removal efficiency of Pb for the composite, 0.85 mm and 0.150 mm soil fractions across the different pulp densities and washing times obtained by the 5% acetic acid and 5% KCL solution. However, a significant difference was observed in percentage removal of Pb within the composite, 0.85 mm and 0.150 mm fractions for 2 to 6 hours extraction with the 5% acetic acid and 10% KCL washing solution. This indicates that the 5% acetic acid and 5% KCL washing solution is likely more efficient for Pb removal. In comparison, the statistical analysis (p=0.05) indicated that there was a significant difference between the percentage removal efficiency of Pb obtained by the two washing solutions when applying similar washing durations of 2, 4, 6, 8 and 10 hours for the different soil fractions. Increasing soil pulp density leads to decrease in Pb removal efficiency for both washing solutions. This may be attributed to possible oversaturation of the washing solution with the soil and the limited amount of potassium chloride available for exchange with Pb within the soil pores system.

**Table 4:** Analysis of Variance on Three Gradation Soil Samples (Composite, 0.85 mm and 0.150 mm) for 3 to 18% Soil Pulp Density

Time interval (hours)	F values	
	5% acetic acid/5% KCL	5% acetic acid/10% KCL
2	0.5851^a^	7.4979 ^b^
4	3.1500 ^a^	8.7637 ^b^
6	0.8941 ^a^	18.929 ^b^
8	0.2293 ^a^	0.8154 ^a^
10	2.0806 ^a^	0.0539 ^a^

^a^ = no significance difference,

^b^ = significance difference.

F critical = 3.6823

Generally, the removal of Pb from the contaminated soils showed higher percentage removal efficiency with 5% acetic acid and 5% KCL compared with the 5% acetic acid and 10% KCL solution. This may be because the extractability of Pb from contaminated soil is greatly influenced by the pH and concentration of the washing fluid. At lower pH (pH of solution = 1.34, with soil = 2.43), the protons (hydron) added from the acetic acid can react with soil surface sites (silicate minerals and functional groups such as Al-hydroxide Fe-hydroxide and carboxyl) and enhance desorption of Pb cations, which are transferred into the washing solutions.^28^ The Pb cation forms a soluble Pb-chloro complex with chloride ions in the solution, while potassium replaces Pb on the reactive surface of the soil matrix. Comparing both washing solutions, it was assumed that a higher percentage of acetic acid (lower pH) is present in the 5% acetic acid and 5% KCL solution as compared with the 5% acetic acid and 10% KCL solution (pH difference of 1.40), which results in greater solubilization of Pb. The high concentration of KCL may be responsible for the lower removal efficiency of Pb for the 5% acetic acid and 10% KCL washing solution. Consequently, about 57% non-detrital Pb (i.e., associated with water soluble, carbonates and organic) was assumed to be removed from the soils, since these fractions are most amenable to metal removal by chemical leaching.^29^ Acetic acid is a weak organic acid, and therefore, due to dissolution effects, the excess KCL in the second washing solution may partially remove Pb from the crystalline lattice of the soil.^5^,^15^

The percentage removal of Pb in the contaminated soil for both washing solutions was observed to be lower than the 97% reported by Guemiza et al. when using 0.125 M sulfuric acid and 4 M NaCL with a pulp density of 10% at 1 hour washing time, although this value was obtained after three successive acid leachings.^30^ However, the result obtained with 5% acetic acid in 5% KCL in this study compares closely with the 75% removal reported by Lafond et al. using 1 M sulfuric acid and 4 M NaCL solution for 10% pulp density, and the 65% removal of Pb using 5.5 mol NaCl/L in 25% (w/w) of soil pulp density maintained at pH 3.0 by Djedidi et al.^11^,^13^ However, in the study by Lafond et al., the results were obtained after just 1 hour of soil washing which may be due to the strong acid used.^11^ Using 0.05 M di-sodium ethylenediaminetetraacetic acid, Demir and Koleli obtained a removal efficiency ranging from 50–70% for just 2 hours of washing, which is also comparable with the results of the present study.^12^ The results of the 5% acetic acid and 5% KCL were higher than the 47% reported by Oustan et al and Kashem et al. for Pb removal in contaminated soils using only acetic acid as a washing solution.^31^,^32^

### Electrochemical Reduction of Lead

In this study, Al-Al and Al-Fe electrode combinations proved efficient for electrochemical removal of Pb from leachate. Increasing the duration of the electrochemical reduction process may possibly further increase the removal efficiency of Pb according to the observed trend. Results obtained compared closely with the 94% Pb removal from acid and saline leachates using mild steel electrodes, 57–76% using −2.0 V potential sources,^15^ but below 99.9% when using Fe mono-polar electrodes at a current intensity of 3.0 A.^13^,^33^

## Conclusions

In conclusion, the lead concentration in soils at the impact berm related positively with the number of corroded bullet fragments in the soil. The concentration of lead was very high in the composited and 2.00 mm soil fraction, which suggests the presence of small weathered fragmented bullet fragments in the soil. The 5% acetic acid and 5% KCL washing solution was the most efficient solution for Pb removal when it was applied with a 3% soil pulp density and 6 hours washing duration. Longer contact times between the soil and washing solution are desirable. In addition, increasing the number of washing steps will further improve removal efficiency. The Al-Al and Al-Fe bipolar electrode combinations showed the best removal efficiency of Pb from the leachates with values well over 90% at current intensities of 7 and 2.5 A. Although acetic acid is known to be less efficient at Pb removal from contaminated soil, the introduction of potassium chloride enhanced the removal efficiency of Pb, and this washing solution may be an alternative to strong acid and chelating agents. The combined 5% acetic acid and 5% potassium chloride washing solution should pose less risk to the soil ecosystem and the environment in general. Further studies are required to determine the effectiveness of much lower strength acetic acid in combination with other salts for lead removal in contaminated soil.
